# Gaussian mixture modeling of acceleration-derived signal for monitoring external physical load of tennis player

**DOI:** 10.3389/fphys.2023.1161182

**Published:** 2023-03-24

**Authors:** Yoshihiro Marutani, Shoji Konda, Issei Ogasawara, Keita Yamasaki, Teruki Yokoyama, Etsuko Maeshima, Ken Nakata

**Affiliations:** ^1^ Graduate School of Sport and Exercise Sciences, Osaka University of Health and Sport Sciences, Kumatori, Osaka, Japan; ^2^ Department of Health and Sport Sciences, Graduate School of Medicine, Osaka University, Toyonaka, Osaka, Japan; ^3^ Department of Sports Medical Biomechanics, Graduate School of Medicine, Osaka University, Suita, Osaka, Japan

**Keywords:** wearable sensor device, exercise intensity, accelerometry, histogram, multimodal distribution

## Abstract

**Introduction:** With the widespread use of wearable sensors, various methods to evaluate external physical loads using acceleration signals measured by inertial sensors in sporting activities have been proposed. Acceleration-derived external physical loads have been evaluated as a simple indicator, such as the mean or cumulative values of the target interval. However, such a conventional simplified indicator may not adequately represent the features of the external physical load in sporting activities involving various movement intensities. Therefore, we propose a method to evaluate the external physical load of tennis player based on the histogram of acceleration-derived signal obtained from wearable inertial sensors.

**Methods:** Twenty-eight matches of 14 male collegiate players and 55 matches of 55 male middle-aged players wore sportswear-type wearable sensors during official tennis matches. The norm of the three-dimensional acceleration signal measured using the wearable sensor was smoothed, and the rest period (less than 0.3 G of at least 5 s) was excluded. Because the histogram of the processed acceleration signal showed a bimodal distribution, for example, high- and low-intensity peaks, a Gaussian mixture model was fitted to the histogram, and the model parameters were obtained to characterize the bimodal distribution of the acceleration signal for each player.

**Results:** Among the obtained Gaussian mixture model parameters, the linear discrimination analysis revealed that the mean and standard deviation of the high-intensity side acceleration value accurately classified collegiate and middle-aged players with 93% accuracy; however, the conventional method (only the overall mean) showed less accurate classification results (63%).

**Conclusion:** The mean and standard deviation of the high-intensity side extracted by the Gaussian mixture modeling is found to be the effective parameter representing the external physical load of tennis players. The histogram-based feature extraction of the acceleration-derived signal that exhibit multimodal distribution may provide a novel insight into monitoring external physical load in other sporting activities.

## Introduction

Wearable sensor devices allowing objective and real-time monitoring are becoming widespread in competitive sports (Patel et al., 2015; Iqbal et al., 2016; Camomilla et al., 2018; Aroganam et al., 2019). Sports scientists, trainers, coaches, and athletes use wearable devices to evaluate physical loads to improve performance and reduce the risk of injury (Cross et al., 2016; Malone et al., 2017; Esmaeili et al., 2018; Duggan et al., 2021). Physical load monitoring indicators have been classified into internal and external physical loads (Halson, 2014). The external physical load is defined as the work completed, as assessed by mechanical indices, whereas internal physical loads are physiological and psychological stresses imposed by external loads, as assessed by heart-rate-based indices, other bio-signals, and questionnaires (Halson, 2014). Excessive external loads resulting from training activities and competitive matches are the major causes of injury in skeletal muscle systems (Gabbett, 2004; Rogalski et al., 2013). Particularly, the frequency of high-intensity acceleration/deceleration movement is associated with subsequent muscular damage and causes a decline in neuromuscular function (Young et al., 2012; Hulin et al., 2016; Russell et al., 2016; Taylor et al., 2018; Gastin et al., 2019). Therefore, external physical loads have been used as an indicator to prevent overload-related injuries during daily training and competition (Gabbett, 2004; Rogalski et al., 2013).

Position-derived evaluation using the global positioning system (GPS) and local positioning system (LPS) have been used to assess external physical loads in various competitive sports (Coutts and Duffield, 2010; Waldron et al., 2011; Cummins et al., 2013; Johnston et al., 2014; Wellman et al., 2016; Mujika, 2017; Linke et al., 2018). The travel distance, speed, and acceleration of the players were evaluated using time-series changes in the player’s position (Johnston et al., 2018; Clemente et al., 2019). However, GPS-based devices have some limitations: they cannot be used indoors, their validity and reliability may decrease when including short-cutting and jumping movements, and the travel distance and speed have been reported to be underestimated (Duffield et al., 2010; Rawstorn et al., 2014; Vickery et al., 2014; Scott et al., 2016). Although LPS acquires position data with higher accuracy than GPS in both indoor and outdoor environments (Hoppe et al., 2018; Alt et al., 2020), the time-consuming set up and calibration of antennas prior to measurement are practical concerns for the daily use of LPS.

The acceleration-derived evaluation of physical loads mitigates the disadvantage of position-derived evaluation of external physical loads (Cambers et al., 2015; Spangler et al., 2018). Wearable and small inertial sensors incorporating acceleration sensors have been used to evaluate the external physical load in competitive matches and daily practice (Sato et al., 2009; Alexander et al., 2016). The inertial sensor can record three-dimensional acceleration at a high sampling frequency over long period (Sato et al., 2009; Wundersitz et al., 2015b; Cambers et al., 2015; Alexander et al., 2016; Spangler et al., 2018). The norm of acceleration or norm of change in acceleration is a major index of acceleration-derived physical loads and has been used as a valid and reliable index (Boyd et al., 2011; Bredt et al., 2020; Byrkjedal et al., 2022). These acceleration-derived physical loads have been evaluated as simple representatives, such as mean or cumulative values across the target interval (Rowlands et al., 2015; Bowen et al., 2017; Staunton et al., 2017; Gentles et al., 2018; Staunton et al., 2018; Reche-Soto et al., 2019).

If the acceleration-derived physical load can be assumed to be normally distributed, the mean value is appropriate as a representative value. In contrast, the distribution of the acceleration-derived physical load in sports that include exercises of various intensities, ranging from walking to sprinting, remains unclear. The mean value may not adequately represent the features of the acceleration-derived external physical load assuming a simple unimodal normal distribution. Therefore, we aimed to propose a method to evaluate acceleration-derived physical loads based on the histogram of the acceleration signal obtained from wearable inertial sensors.

## Materials and methods

### Participants

The participants of this study were 14 male collegiate (age: 19.5 ± 1.4 years; height: 168.6 ± 1.4 cm; body mass: 63.2 ± 3.5 kg) and 55 male middle-aged players (age: 54.8 ± 8.7 years; height: 171.1 ± 6.0 cm; body mass: 69.3 ± 9.9 kg) who participated in regional qualifier rounds for national tennis championships in their categories (intercollege championship and master’s championship) (Table1). All participants provided written informed consent, and the study was approved by the institutional review board (19537-2).

**TABLE 1:** Descriptive statistics of participant’s age, body height, and body mass. Data was presented as mean and standard deviation.

Category	Collegiate (*n* = 14)	Middle-aged		
		40s (*n* = 22)	50s (*n* = 15)	60s (*n* = 18)
Age (years)	19.5 ± 1.4	47.1 ± 1.7	51.8 ± 1.4	66.6 ± 1.6
Body height (cm)	168.6 ± 3.8	172.9 ± 5.7	172.9 ± 6.5	167.5 ± 4.3
Body mass (kg)	63.2 ± 3.5	70.7 ± 9.5	73.8 ± 10.8	63.9 ± 7.4

### Data collection

Each participant wore a spandex sportswear-type sensing wear (sportswear-type wearable) particularly designed for physical activity measurement (MATOUSVS, Teijin Frontier Sensing Ltd., Osaka, Japan). The sensing unit, comprising an inertia sensor and a data logger (SS-ECGHRAG, Sports Sensing Ltd, Fukuoka, Japan), was securely fixed at the upper back of the sensing wear. Before data measurement, because we confirmed that the size of the sensing wear was properly adjusted to the upper body of each participant, the acceleration data measured with the fixed inertia sensor accurately reflected the participant’s movement. In addition, the feasibility of this sensing wear to record exercise intensity has been validated (Marutani et al., 2022). In total, 28 and 55 singles matches were recorded from collegiate and middle-aged plyers, respectively. The three-dimensional acceleration signals for 22 games of collegiate players were recorded at 1,000 Hz, and the data for 6 games of collegiate players, as well as 55 games of middle-aged players were recorded at 200 Hz. The processed data (Supplementary Material) was not influenced by the difference in sampling frequency.

### Proposed method

**FIGURE 1:**
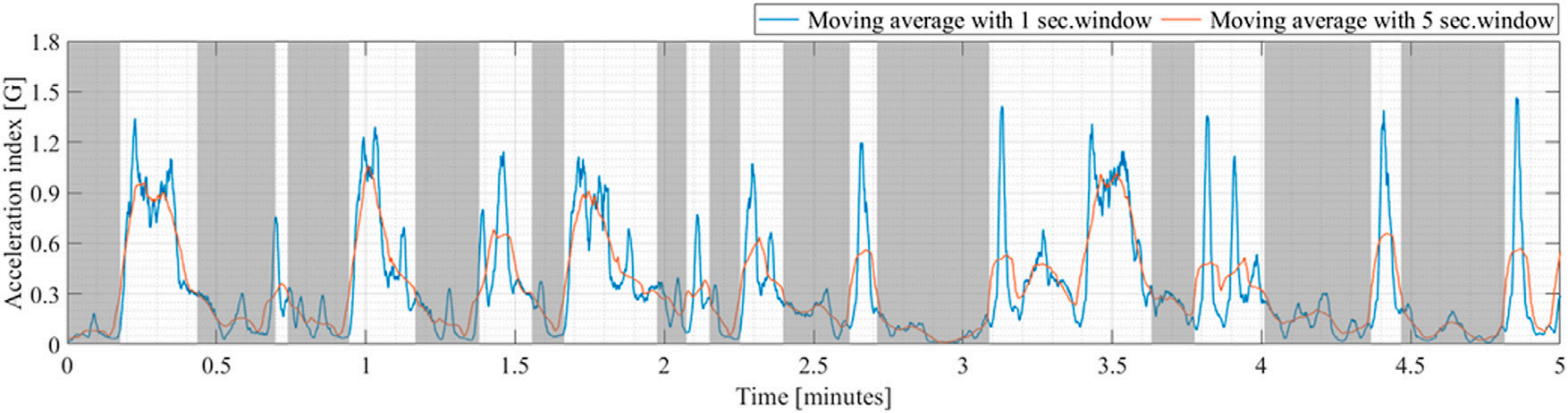
Time-series change in acceleration index (blue) and classification of in-play (white) and rest interval (gray) based on the processed acceleration (orange). Data of 5 min was extracted from approximately 1-hour data of a competitive tennis match to visualize in-play and rest intervals.

The recorded three-dimensional acceleration signal was filtered using a second-order Butterworth band-pass filter (0.5–20 Hz) to remove the high-frequency noise and baseline shift owing to the long-time recording, which also attenuated the gravity component from the acceleration signals. The cut-off frequency was determined with reference to previous studies using trunk mounted inertia sensor for evaluating locomotive motion (walking, jogging, and running), team sports, and contact sports (Wundersitz et al., 2015a; Wundersitz et al., 2015b; Wundersitz D. W. T. et al., 2015). After calculating the norm of the filtered acceleration signal ( $\left.\left|a\right|=\sqrt{a_{x}^{2}+a_{y}^{2}+a_{z}^{2}}\right)$ , a moving average filter with 1-s window was used. The obtained smoothed absolute acceleration signal is referred to as the acceleration index (Ward et al., 2016) and is used as the main signal for the Gaussian mixture model fitting (blue line in Figure 1). To identify the player’s resting period during the competitive match, such as point-to-point and game-to-game intervals, we identified the period where the value of the smoothed signal (moving average with a 5-s window represented by the orange line in Figure 1) was less than 0.3 G for at least 5 s and defined the period as the rest interval. The period, rather than the rest interval, was defined as in-play (Figure 1). We assumed that the low intensity movement during in-play rally is unlikely to continue for more than 5 s. Using this cut-off value, the video image of the match confirmed that the in-play and rest intervals were classified almost exactly.

**FIGURE 2:**
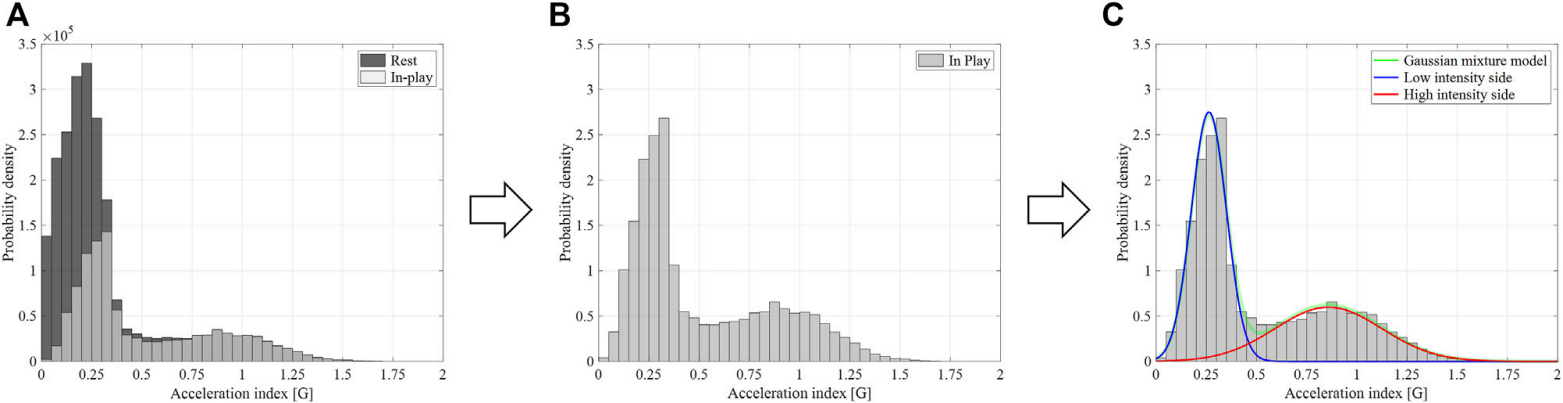
Process of Gaussian mixture modeling: **(A)** Histogram of the acceleration index during the classified in-play and rest intervals, **(B)** Histogram of the acceleration index during the extracted in-play interval, **(C)** Fitted bimodal Gaussian mixture model.

The histogram of the acceleration index during the in-play showed a meaningful bimodal distribution, which comprised a low-intensity peak that appeared at approximately 0.25 G and a high-intensity peak at approximately 0.9 G in the representative data (Figure 2). To characterize this meaningful distribution of the in-play acceleration index value, a Gaussian mixture model was fitted to the histogram of the acceleration index during the in-play (Figure 2).

$$p\left(x\right)=\sum_{k=1}^{2}w_{k}N\left(x|\mu_{k},\sigma_{k}^{2}\right)$$ (1)

$$N\left(x|\mu_{k},\sigma_{k}^{2}\right)=\frac{1}{\sqrt{{2\pi \sigma }_{k}^{2}}}\exp\left\{-\frac{1}{{2\sigma }_{k}^{2}}{\left(x-\mu_{k}\right)}^{2}\right\}$$ (2)

where p(x) is the resultant mixture of two probability density functions of the normal distributions $N\left(x|\mu_{k},\sigma_{k}^{2}\right)$ . The mixing ratio $w_{k}$ determines the weight of each probability density. The parameters μ ; $\sigma^{2}$ in the probability density function of the normal distribution are the mean and standard deviation, respectively. In the model fitting process, the parameters ( $\mu_{1},\mu_{2},\sigma_{1},\sigma_{2},w_{1},{and\;w}_{2}$ ) were estimated using the maximum likelihood estimation of the mixed normal distribution model using the EM algorithm for the acceleration index (***x*)**. Because the mixing rate satisfies $w_{1}+w_{2}=1.0$ , five parameters ( $\mu_{low},\sigma_{low},\mu_{high},\sigma_{high},{and\;w}_{high}$ ) are used as the features obtained from the proposed method in this study.

### Comparison of proposed and conventional methods

The proposed and conventional methods were compared to validate the effectiveness of the proposed method. In previous studies, the norm of the raw acceleration signal was calculated, and the mean value across the target period ( $\mu_{total}$ ) was used as the feature. The features obtained using the proposed and conventional methods were standardized to have a mean of 0.0 and a standard deviation of 1.0 for comparison. As the proposed method has five features, the distribution of each feature and the relationship between features were visualized using a scatterplot matrix. We assumed that the intensity of play is seems to be differ between collegiate and middle-aged players based on the observation of tennis matches; therefore, we searched for a combination of features that could clearly show this group difference compared with conventional methods. The statistical distance between the means of each group in the feature space was calculated to evaluate the effectiveness of the proposed method compared with conventional methods. The discrimination accuracy of the two groups (collegiate and middle-aged players) based on the features obtained using the proposed and conventional methods was validated using linear discriminant analysis. All data analyses were performed using the custom-made MATLAB R2020a program (MathWorks, Inc., United States).

## Results

**FIGURE 3:**
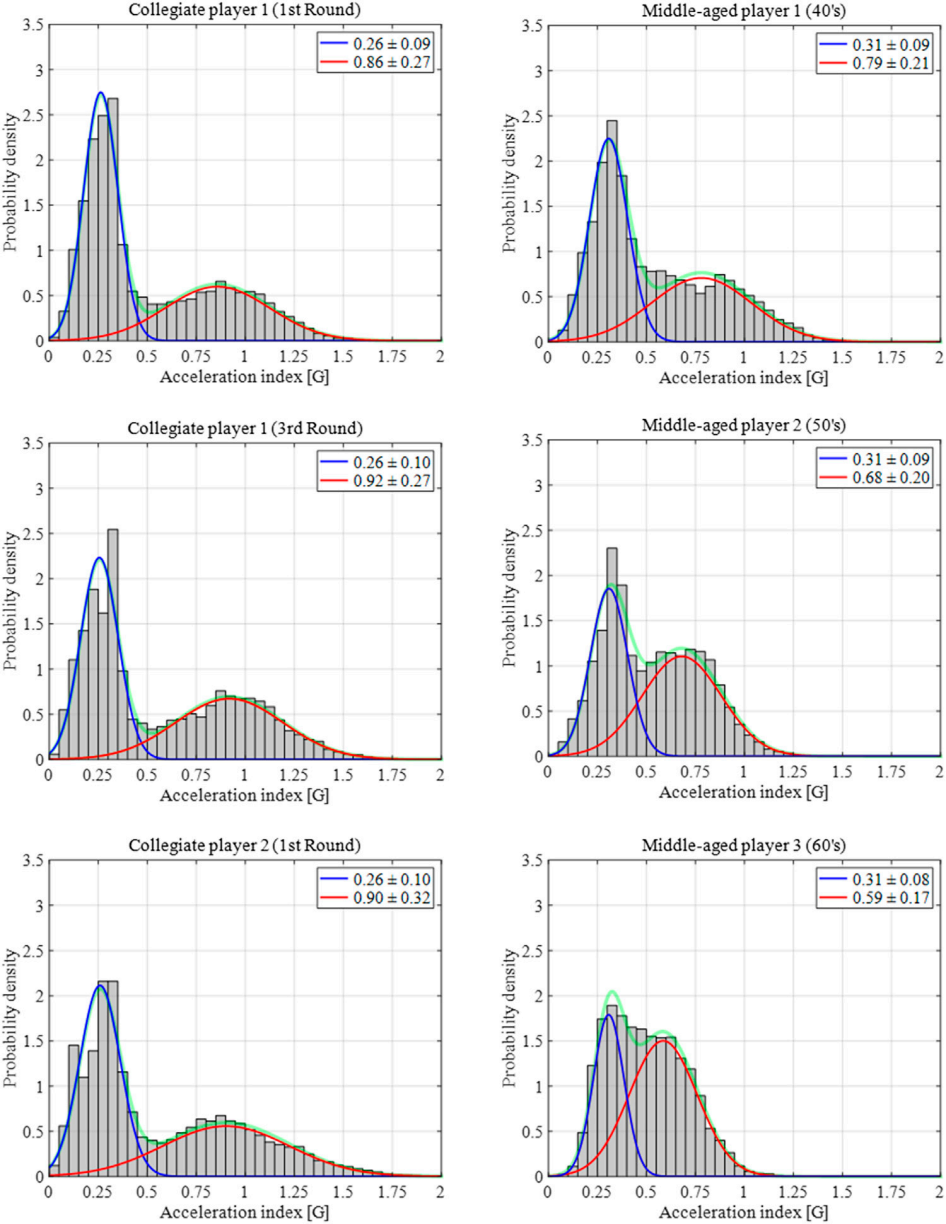
Intra- and inter-subject differences, as well as inter-group differences of the Gaussian mixture model on the histogram of the acceleration index. The green line denotes the fitted Gaussian mixture model comprising the high- (red) and low-intensity (blue) sides.

Eighty-three games played by collegiate and middle-aged players revealed that the acceleration index during in-play showed a bimodal distribution, which was approximated using a Gaussian mixture model comprising two normal distribution models (Figure 3). The intra-subject, inter-subject, and inter-group differences in the Gaussian mixture model on the histogram of the acceleration index are shown in Figure 3.

**FIGURE 4:**
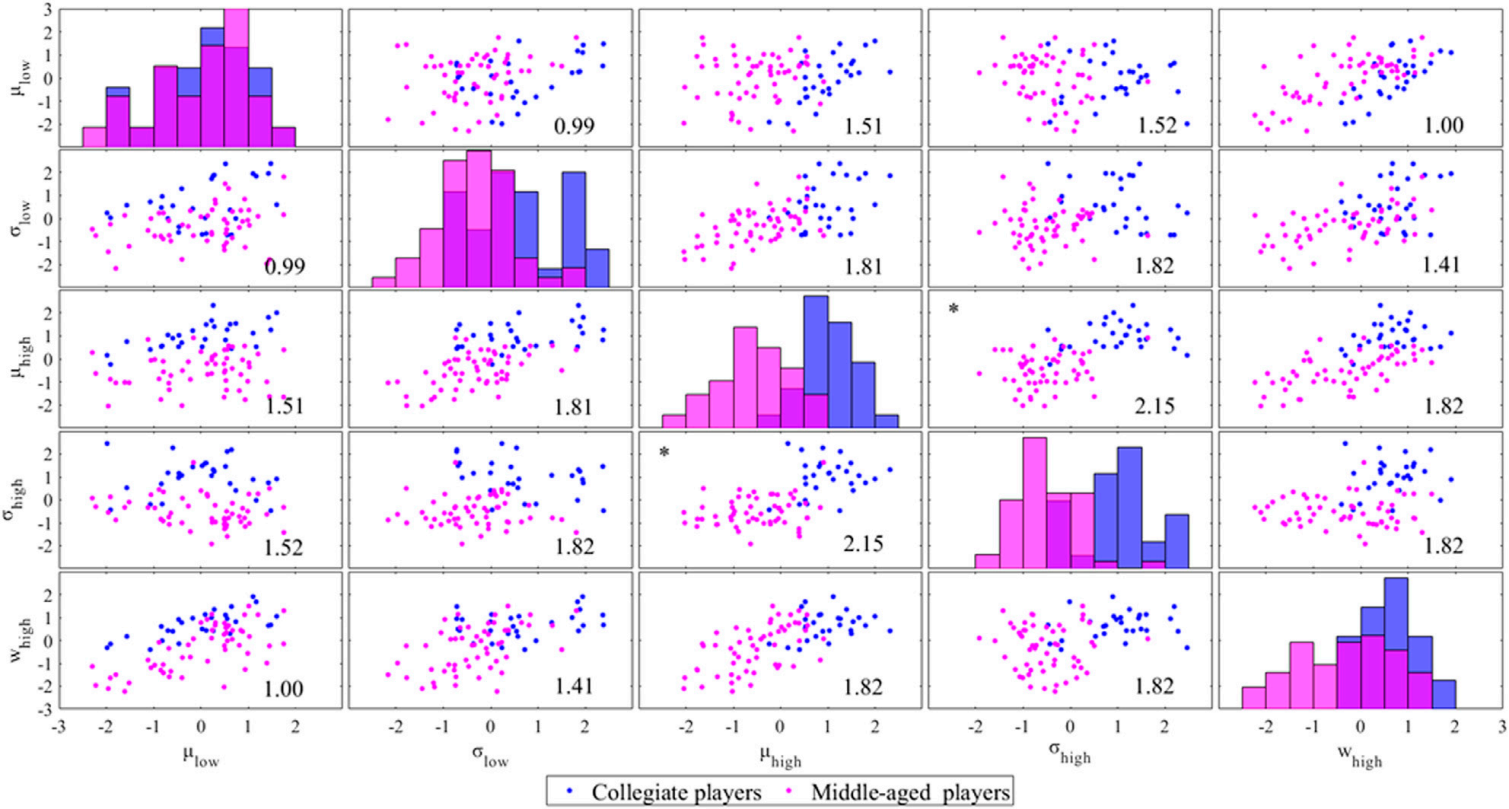
Scatter matrix of parameters of the bimodal Gaussian mixture model labeled by two groups. The parameters were standardized by the mean and standard deviation. The number in each panel shows the Euclidean distance between the mean values of two groups. The asterisks (*) shows the combination of parameters with the greatest Euclidean distance.

The mean ( $\mu_{low}$ ), standard deviation ( $\sigma_{low}$ ), and parameters of the normal distribution on the low-intensity side of the acceleration index showed no inter-group differences (Figure 4). Conversely, for the parameters of the normal distribution on the high-intensity side of the acceleration index, mean ( $\mu_{high}$ ), and standard deviation ( $\sigma_{high}$ ), inter-group differences were evident (Figure 4). The combination of the mean ( $\mu_{high}$ ) and standard deviation ( $\sigma_{high}$ ) of the normal distribution of the high-intensity side showed the largest Euclidean distance between the means of the collegiate and middle-aged players.

**FIGURE 5:**
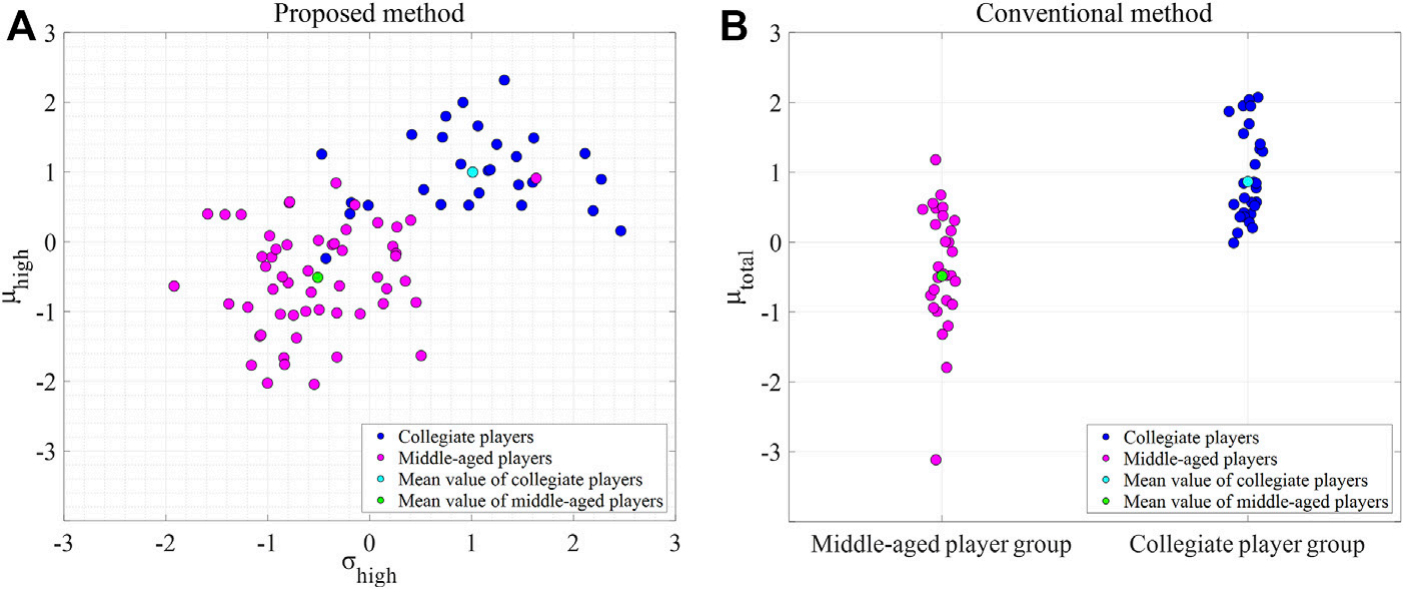
Comparison between the proposed **(A)** and conventional method **(B)**. The proposed method used the mean and standard deviation of the high-intensity side. Each parameter was standardized using the mean and standard deviation.

The Euclidean distance in the plane composed of the mean ( $\mu_{high}$ ) and standard deviation ( $\sigma_{high}$ ) of the high-intensity side was 2.15, compared with 1.43 when only the mean ( $\mu_{total}$ ) of the overall distribution was employed using the conventional method (Figure 5). The classification accuracy of the linear discriminant analysis was 93% for the proposed method using the mean ( $\mu_{high}$ ) and standard deviation ( $\sigma_{high}$ ) of the normal distribution, indicating the high-intensity side, and 63% for the conventional method using only the overall mean ( $\mu_{total}$ ).

## Discussion

We proposed a method to evaluate the external physical load based on the histogram of the acceleration index recorded by wearable sensors. The histogram of the acceleration index during the competitive tennis match showed a bimodal distribution that could be modeled using a Gaussian mixture model. The mean ( $\mu_{high}$ ) and standard deviation ( $\sigma_{high}$ ) of the high-intensity side in the Gaussian mixture model were potential parameters for characterizing the difference in the external physical load between the player groups (collegiate and middle-aged). When these variables were used as features, the classification accuracy was higher than that of conventional methods in classifying two groups with different intensities during competitive tennis matches (Figure 5). Based on the results, the mean and standard deviation of the high-intensity side of the Gaussian mixture model derived from the acceleration index are effective in monitoring external physical loads in competitive sports, including exercises of various intensities, ranging from standing and walking to sprinting.

The histogram of the acceleration index during the competitive tennis match showed a bimodal distribution and was adequately approximated using the bimodal Gaussian mixture model (Figure 3). Our previous study conducted an incremental loading test on a treadmill to examine the change in the acceleration index from quiet standing to sprinting (Marutani et al., 2022). Interpreting the results of our previous study in the context of the present result, we found that the peak of distribution of low- and high-intensity sides were approximately equivalent to the walking and jogging, respectively, and the high-intensity side is widely distributed from the jogging to running and sprinting (Marutani et al., 2022). We speculate that the low-intensity activity may represent the waiting for the opponent to hit during a rally, whereas the high-intensity side may represent the ball chasing and swing movements. A bimodal distribution was observed in the histogram of collegiate players’ acceleration index compared with that of middle-aged players. The histogram of $\mu_{high}$ and $\sigma_{high}$ became closer to the low-intensity side in middle-aged players, demonstrating a decrease in high-intensity activity. Thus, visualization of the histogram of the acceleration index may be useful in monitoring external physical loads, enabling the application of other sporting activities in practice and competition.

The mean ( $\mu_{high}$ ) and standard deviation ( $\sigma_{high}$ ) of the high-intensity side of the bimodal Gaussian mixture model differed between collegiate and middle-aged tennis players (Figure 4). The distance between the two groups was the largest when using the combination of $\mu_{high}$ and $\sigma_{high}$ (Figure 4), and the linear discriminant analysis showed a higher accuracy compared with the conventional method using only the overall mean of the acceleration index (Figure 5). We suggested that the mean $\mu_{high}$ and standard deviation $\sigma_{high}$ of the high-intensity peak were sensitive features for characterizing the external physical load during a tennis match. In contrast, the parameters of the low-intensity peak ( $\mu_{low}$ ; $\sigma_{low}$ ) were similar between the collegiate and middle-aged players (Figure 4), demonstrating that the low-intensity movements are commonly included during in-play in the collegiate and middle-aged players. By excluding the influence of common features of the low-intensity side across players, we extracted highly sensitive parameters representing the distribution of the high-intensity side. Acceleration-derived external physical loads were decreased by intermittent exercise-induced fatigue, and a 15% reduction in acceleration-derived external physical loads during the match when tennis matches were played for four consecutive days has been reported (Reid and Duffield, 2014; Ward et al., 2016; Beato et al., 2019; Truppa et al., 2020). Using the proposed method, the time-series change in the external physical load can be more sensitive, and it is expected to monitor players’ conditions. The proposed method may be sensitive to differences in the external physical load of the players during matches and practices, and it is to be expected to monitor the players’ condition and quality of matches and practice.

This study has two major limitations. First, the proposed method was only tested for competitive tennis matches. However, we believe that the proposed method to evaluate the external physical load based on the distribution of the acceleration index applies to other sporting activities during competition and training. Second, the tested dataset was only recorded from male tennis players because of the structural limitation of sensing wear. However, if a sportswear-type wearable device designed for women can record the acceleration signal using a trunk-mounted inertia sensor, the shape feature of bimodal distribution of female players would show a similar trend to male players. However, its intensity of female players is expected to be different from that of male players.

## Conclusion

We proposed a method to evaluate the external physical load based on the histogram of the acceleration index recorded by wearable sensors. The mean and standard deviation of the high-intensity side extracted by the Gaussian mixture modeling are found to be the effective parameter representing the external physical load of tennis players. The histogram-based feature extraction of the acceleration-derived signal that exhibit the multimodal distribution may provide a novel insight into monitoring external physical load in various sporting activities.

## Data Availability

The raw data supporting the conclusion of this article will be made available by the authors, without undue reservation.
