# Supplementary material for: Gaussian mixture modeling of acceleration-derived signal for monitoring external physical load of tennis player
Source: Front Physiol. 2023 Mar 24;14:1161182. doi: 10.3389/fphys.2023.1161182 (PMC10079886; doi:10.3389/fphys.2023.1161182)
Supplement: Supplementary file 1 [file Image1.pdf]

Supplementary Material

To validate the difference in sampling frequency, a continuous swing motion with sidesteps imitating the tennis stroke was recorded by two inertial sensors operating at 200 and 1000 Hz. The time-series change (upper panels) and histogram (bottom panels) of the acceleration index (processed signal described in the Methods section) were similar between the two sampling frequencies (Figure S1).

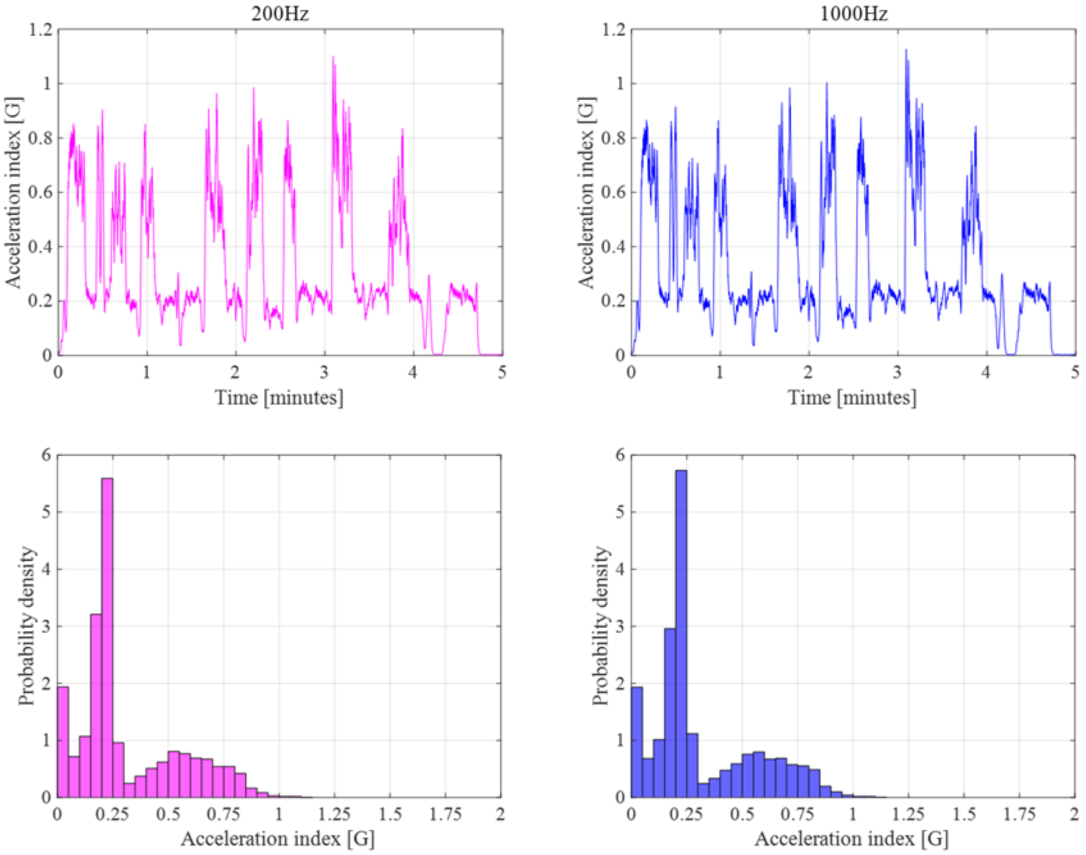

Figure S1. Accerelation indices and histograms from the difference sampling frequency sensors.
